# Gout incidence in metformin versus sodium–glucose co-transporter-2 inhibitor users: a retrospective cohort study

**DOI:** 10.1093/rheumatology/keaf136

**Published:** 2025-03-24

**Authors:** Masaki Hatano, Akira Okada, Yusuke Sasabuchi, Hisatoshi Ishikura, Takeyuki Tanaka, Taku Saito, Sakae Tanaka, Hideo Yasunaga

**Affiliations:** Department of Clinical Epidemiology and Health Economics, The University of Tokyo, Tokyo, Japan; Department of Orthopaedic Surgery, The University of Tokyo, Tokyo, Japan; Department of Prevention of Diabetes and Lifestyle-Related Diseases, The University of Tokyo, Tokyo, Japan; Department of Real-world Evidence, The University of Tokyo, Tokyo, Japan; Department of Orthopaedic Surgery, The University of Tokyo, Tokyo, Japan; Department of Orthopaedic Surgery, The University of Tokyo, Tokyo, Japan; Department of Orthopaedic Surgery, The University of Tokyo, Tokyo, Japan; Department of Orthopaedic Surgery, The University of Tokyo, Tokyo, Japan; Department of Clinical Epidemiology and Health Economics, The University of Tokyo, Tokyo, Japan

**Keywords:** gout, cohort studies, diabetes mellitus, hyperuricaemia, hypoglycaemic agents, metformin, sodium–glucose co-transporter-2 inhibitors

## Abstract

**Objectives:**

To compare the incidence of gout in individuals with diabetes receiving metformin *vs* sodium–glucose co-transporter-2 inhibitors (SGLT-2is).

**Methods:**

This new-user comparative effectiveness study included adults from the JMDC claims database with health check-up and administrative claims data from 2014 to 2022. Individuals initiated on metformin were compared with those initiated on SGLT-2is. The primary outcome was the incidence of new gout diagnoses. After propensity-score inverse probability treatment weighting (IPTW), Cox proportional hazards models were fitted to estimate the hazard ratios (HRs) and 95% CIs. A linear mixed model was employed to assess the association between the two groups and changes in serum uric acid levels.

**Results:**

A total of 21 561 individuals with diabetes were identified, including 17 636 males with a mean age of 53 years. The metformin and SGLT-2i groups included 13 535 and 8026 individuals, respectively. In the metformin and SGLT-2i groups, the gout incidence rates were 2.40 and 3.15 events per 1000 person-years, respectively. After IPTW, metformin was not associated with a decreased risk of gout compared with SGLT-2i (HR 0.90, 95% CI 0.63–1.28; rate difference –0.28, 95% CI –1.24 to 0.68 events per 1000 person-years). The mean difference in serum uric acid level change 1 year after the index date was 0.48 mg/dl (95% CI 0.43–0.52) for the metformin group relative to the SGLT-2i group.

**Conclusion:**

Gout risk may be comparable between metformin and SGLT-2is in individuals with diabetes, with metformin showing a lesser reduction in serum uric acid levels than SGLT-2is.

Rheumatology key messages

- Metformin and sodium–glucose co-transporter-2 inhibitors may be viable options in diabetes treatment when considering gout risk.
- Metformin was less effective in reducing serum uric acid levels.

## Introduction

Gout is a common chronic disorder that results from monosodium urate crystal deposition and is characterized by acute inflammatory arthritis [1]. It correlates with increased risks of disability, cardiovascular disease and mortality [2]. Gout development is attributed to multiple factors, including hyperuricaemia, post-traumatic events, obesity, and metabolic disorders, such as diabetes [1, 3]. Therefore, treatment strategies for diabetes should consider addressing the risk of gout to potentially improve health outcomes.

In 2022, the American Diabetes Association recommended sodium-glucose co-transporter-2 inhibitors (SGLT-2is) as initial therapy for type 2 diabetes in individuals at high risk of atherosclerotic cardiovascular disease, heart failure and/or chronic kidney disease [4]. Growing evidence shows that SGLT-2is have various effects beyond lowering serum glucose levels [5]. These effects lead to cardiovascular or renal benefits in individuals with and those without diabetes [6]. Recent meta-analyses suggest that SGLT-2is lower serum uric acid levels by increasing urinary uric acid excretion [7]. Moreover, recent epidemiological studies have shown the superiority of SGLT-2is over other antihyperglycaemic agents, such as glucagon-like peptide-1 receptor agonists, dipeptidyl peptidase-4 inhibitors and sulfonylureas, in reducing the risk of gout [8–11].

Metformin has historically been the first-line oral antihyperglycaemic agent and remains widely used. It possesses direct anti-inflammatory effects in addition to improving hyperglycaemia [12–15]. A recent study indicates that metformin use is associated with a lower risk of gout among individuals with pre-diabetes, although its effectiveness in the broader population with diabetes remains unknown [16]. Furthermore, whether metformin or SGLT-2i use is more effective in reducing the risk of gout remains uncertain. This gap in evidence contributes to uncertainties in decision-making regarding the choice of antihyperglycaemic agents for individuals with diabetes, considering their risk of gout.

In Western countries, where the indications for metformin and SGLT-2is have long differed, comparing the two may be challenging. No specific class of antihyperglycaemic agents is consistently selected as the first-line treatment for type 2 diabetes in Japan [17]. Therefore, population-based studies in this context may provide comparability between metformin and SGLT-2is.

To address this critical knowledge gap, we conducted a retrospective cohort study to compare the risk of gout in individuals with diabetes who initiated metformin *vs* SGLT-2is, using a nationwide administrative claims database with health check-up data, enabling a long-term follow-up.

## Methods

### Study design and setting

This retrospective cohort study used the JMDC claims database (Tokyo, Japan), which is detailed elsewhere [18]. In summary, the database contains administrative claims submitted to health insurers by clinics, hospitals and pharmacies from April 2007 to 31 May 2022. It includes information from multiple in-country organizations providing health insurance to employees and their families and contains data on annual health check-ups for lifestyle-related diseases [19]. Health check-up data include the date, participants’ demographics (including age, sex, BMI and blood pressure), laboratory tests and lifestyle factors collected via questionnaires (e.g. smoking and alcohol habits). Serum uric acid levels can be included in health check-ups if employers or insurers incorporate them as part of routine health monitoring programs for employees and insured individuals [20]. These health check-ups are typically provided as part of company welfare programmes and include mandatory annual check-ups required by the Industrial Safety and Health Act.

Diagnoses are recorded in the JMDC database based on the International Classification of Diseases, 10th Revision (ICD-10) codes. Prescribed drugs and procedures are recorded based on the World Health Organization Anatomical Therapeutic Chemical Classification System (WHO-ATC) and Japanese procedure codes, respectively.

This study was approved by the Institutional Review Board at the University of Tokyo [approval number: 10862–(1)]. The requirement for informed consent was waived because all data were de-identified.

### Study participants

To minimize biases inherent in observational studies, we conducted an active comparator, new-user cohort study comparing adults (age ≥18 years) with diabetes who started metformin *vs* those who initiated SGLT-2is, based on their first glycated haemoglobin (HbA1c) level of ≥6.5% (Supplementary Fig. S1, available at *Rheumatology* online). Individuals aged ≥18 years who initiated their first-ever use of metformin or SGLT-2i between 1 April 2014 and 31 May 2022 were eligible for inclusion in this study. The index date was defined as the earliest first-ever prescription date of the drug of interest within the study period. All individuals were required to have undergone a health check-up within 12 months before the index date. A 12-month baseline period was used to assess eligibility for cohort entry, specifically defined as the 12 months before the index date. The exclusion criteria were (i) lack of a 12-month baseline period; (ii) simultaneous initiation of both study drugs; (iii) history of gout diagnosis during the baseline period; (iv) history of type 1 diabetes diagnosis during the baseline period; (v) history of severe renal impairment (including those undergoing maintenance dialysis or renal transplantation) during the baseline period; (vi) use of antigout preparations during the baseline period (using WHO-ATC code); and (vii) lack of follow-up data. Supplementary Tables S1 and S2, available at *Rheumatology* online present the ICD-10 and WHO-ATC codes for the exclusion criteria, respectively.

### Exposure and comparison

The exposure and control groups included individuals initiating prescriptions for metformin (using WHO-ATC codes, A10BA02) (metformin group) and SGLT-2i (A10BK) (SGLT-2i group), respectively, during the study period. Drug use was defined using an observational analogue of the initial treatment approach. Individuals were categorized into the metformin or SGLT-2i group based on the first drug initiated, ensuring that all individuals were new users at the start of the study and minimizing biases associated with prior exposure to the study drugs. Follow-up continued throughout the study period without censoring, even if individuals discontinued the initial study drug, switched to the other study drug, or added the other study drug during the follow-up. The follow-up period ended when the study endpoint occurred, upon death, or at the end of the study period (31 May 2022), whichever came first.

### Outcomes and covariates

The primary outcome was a new diagnosis of gout, whereas the secondary outcome was the change in serum uric acid levels (first to second health check-up). Data for the second health check-up were collected within 1–2 years after the index date to obtain the change in serum uric acid level. The outcome was defined as a diagnosis of gout (using the ICD-10 codes, M10) and prescriptions (using the WHO-ATC codes) for IA or oral CS, colchicine or NSAIDs dispensed within 2 weeks, or hospitalization for gout [10, 21]. Supplementary Table S3, available at *Rheumatology* online presents the WHO-ATC codes for the outcome.

The following information was obtained from the first health check-up: age, sex, BMI, blood pressure, smoking status (current or non-current/never), alcohol consumption (daily or not) and baseline laboratory values (triglycerides, high-density lipoprotein cholesterol, low-density lipoprotein cholesterol, γ-glutamyl transpeptidase, HbA1c, uric acid and proteinuria). Proteinuria was subdivided into five categories as follows: <5, 5–29, 30–99, 100–299 and ≥300 mg/dl.

Diagnostic information was collected during the baseline period, including each component of the Charlson Comorbidity Index [22] and conditions such as hypertension, dyslipidaemia, heart failure, atrial fibrillation, venous thromboembolism, chronic kidney disease, chronic obstructive pulmonary disease, septic arthritis, pseudogout, trauma and RA. The ICD-10 codes for covariates, excluding Charlson Comorbidity Index components, are listed in Supplementary Table S4, available at *Rheumatology* online.

During the 3 months before the index date, prescription history information was obtained for insulin, dipeptidyl peptidase-4, glucagon-like peptide-1, sulfonylureas, alpha-glucosidase inhibitors, thiazolidinediones, glinides, antiplatelet drugs, anticoagulant drugs, cardiac glycosides, antiarrhythmic drugs, beta-blockers, calcium channel blockers, angiotensin-converting enzyme inhibitors/angiotensin receptor blockers, antihyperlipidaemic drugs, endocrine therapy, diuretics, immunosuppressants, NSAIDs and CS. Supplementary Table S5, available at *Rheumatology* online lists the WHO-ATC codes for these covariates.

### Statistical analyses

Data are summarized as medians with interquartile ranges or means with s.d. for continuous variables and as numbers and proportions for categorical variables. Participant demographics were reported for the treatment groups. We conducted a time-stratified, propensity-score inverse probability treatment weighting (IPTW) cohort study to adjust for secular trends in prescription patterns and clinical care [9]. Accordingly, we segmented calendar time into 1-year intervals. Individuals were assigned to these intervals based on their index date, which corresponded to the initiation of metformin or SGLT-2is. In each time block of drug initiation, propensity scores were calculated using logistic regression, with metformin prescription as an outcome and covariates including age, sex, BMI, blood pressure, smoking status, alcohol consumption, baseline laboratory values, Charlson Comorbidity Index score, and diagnostic and prescription history. Stabilized IPTW estimated the average treatment effect on the population [23]. The balance of these characteristics was assessed using standardized mean difference (SMD), with an SMD of >0.1 indicating considerable imbalance between the metformin and SGLT-2i groups. As detailed in Supplementary Table S6, available at *Rheumatology* online, the proportion of missing data was 9.5%. Complete case analysis was used, as <10% of data were missing. The impact of missing data on the main analyses was likely minimal in this study [24].

Crude gout incidence rates were calculated by dividing the total number of gout incidence events by the accumulated person-time from the observational period for each group. The association between the two groups and study outcomes were estimated by calculating crude and weighted hazard ratios (HRs) and 95% CIs from Cox proportional hazards models. Schoenfeld residual test was used to assess the proportional hazards assumption. A weighted cumulative incidence curve was also plotted for gout. Subsequently, the 95% CIs of the incidence rate difference were estimated by bootstrapping using 1000 iterations, with the SGLT-2i group serving as the reference group.

We estimated the association between the two groups and changes in serum uric acid levels in a subset of individuals with non-missing serum uric acid data from the second health check-up. A linear mixed model with an unstructured variance–covariance matrix was used to model uric acid level changes with a random intercept for each person and a fixed effect for metformin use. Serum uric acid levels at the first and second health check-ups were subsequently estimated using marginal standardization [25].

Subgroup analyses were performed for the primary outcome stratified by sex, BMI (<25 *vs* ≥25 kg/m^2^), baseline serum uric acid level (<7.0 *vs* ≥7.0 mg/dl) and baseline HbA1c level (<7.5% *vs* ≥7.5%). *P*-values for interactions were calculated by including an interaction term between a subgroup variable and the exposure drug in the Cox proportional hazards models.

Three sensitivity analyses were conducted to confirm the robustness of our findings. First, we evaluated the association of metformin and SGLT-2is with the primary outcome using an observational approach analogous to the as-treated approach. Individuals were followed up until the study endpoint occurrence, treatment discontinuation, receipt of the other study drug, death or 31 May 2022, whichever occurred first. Treatment discontinuation was defined as the absence of drug dispensing within 60 days of the next expected prescription date. This expected date was determined by adding the days’ supply to the most recent dispensing date of the study drug. Individuals who discontinued the study drug were monitored until the last expected next prescription date, extended by a 60-day grace period. Receipt of the other study drug resulted in immediate censoring. Second, multiple imputation analyses were conducted to test whether missing data influenced the primary outcome [26]. Third, the robustness of our findings was estimated by re-analysing the outcomes using propensity-score overlap weighting instead of IPTW [27]. All statistical analyses were performed using Stata version 18 (Stata Corp, College Station, TX, USA).

## Results

### Participant characteristics

**Figure 1.**
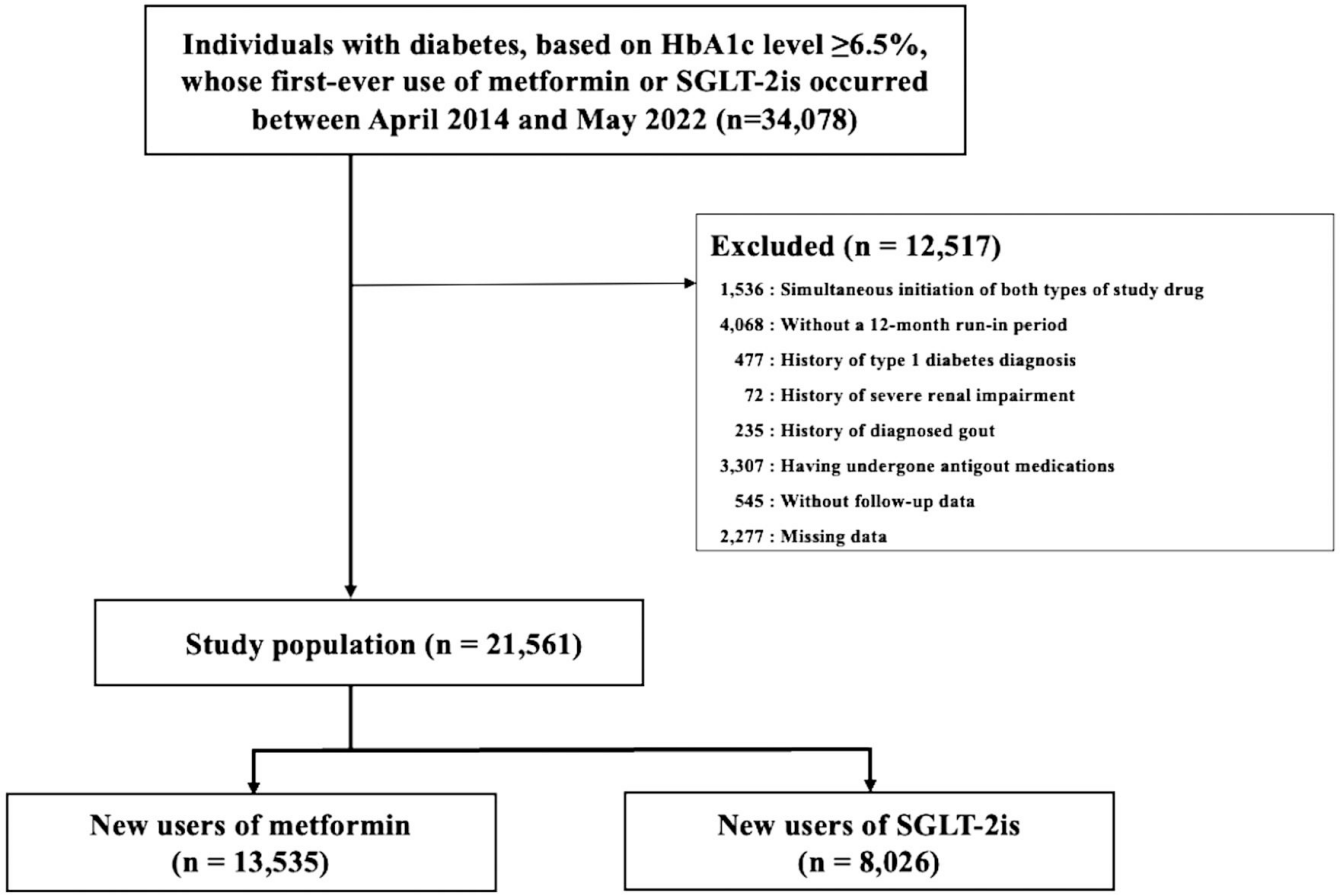
Study flow chart. SGLT-2i: sodium–glucose co-transporter-2 inhibitor

The overall cohort included 21 561 individuals who underwent their first health check-up between 1 April 2014 and 31 May 2022. Of these, 13 535 and 8026 were in the metformin and SGLT-2i groups, respectively, at the index date (Fig. 1). Table 1 presents the baseline personal and health characteristics of both groups before IPTW. Overall, the median age was 52.6 (s.d. 8.5) years and 17 636 (81.8%) were males. The mean follow-up time was 2.7 (s.d. 2.0) and 2.2 (s.d. 1.8) years in the metformin and SGLT-2i groups, respectively. Furthermore, the mean treatment duration was 2.1 (s.d. 1.9) and 1.9 (s.d. 1.6) years in the metformin and SGLT-2i groups, respectively. Supplementary Table S7, available at *Rheumatology* online presents the distribution of the censoring events. A negligible imbalance (SMD <0.1) was observed between the groups after weighting (Table 1).

**Table 1.** Characteristics of individuals who initiated metformin or SGLT-2is before and after IPTW

	Before IPTW			After IPTW		
	Metformin group	SGLT-2i group	SMD	Metformin group	SGLT-2i group	SMD
Total participants, *n*	13 535	8026		13 504	7985	
Mean age, years (s.d.)	52.5 (8.5)	52.8 (8.5)	–0.037	52.6 (8.6)	52.6 (8.4)	0.003
Male sex, *n* (%)	11 044 (82)	6592 (82)	–0.014	11 054 (82)	6540 (82)	–0.001
Mean BMI, kg/m^2^ (s.d.)	27.3 (4.7)	28.4 (4.9)	–0.227	27.7 (4.9)	27.7 (4.7)	–0.002
Smoking status (current smoker), *n* (%)	4917 (36)	2778 (35)	–0.036	4809 (36)	2842 (36)	0.000
Alcohol consumption (daily), *n* (%)	2869 (21)	1739 (22)	0.011	2899 (21)	1692 (21)	–0.007
Mean systolic blood pressure, mmHg (s.d.)	131.6 (17.3)	132.6 (17.1)	–0.06	132.0 (17.3)	132.0 (17.1)	0.002
Mean uric acid, mg/dl (s.d.)	5.5 (1.3)	5.7 (1.3)	–0.166	5.6 (1.3)	5.6 (1.3)	–0.007
Mean HDL-C, mg/dl (s.d.)	51.6 (13.1)	51.3 (12.8)	0.024	51.5 (13.0)	51.5 (13.0)	–0.001
Mean triglycerides, mg/dl (s.d.)	181.5 (150.8)	179.4 (141.8)	0.014	180.7 (147.1)	180.4 (143.5)	0.002
Mean LDL-C, mg/dl (s.d.)	133.7 (34.5)	131.4 (34.0)	0.068	132.8 (34.5)	133.0 (34.0)	–0.004
Mean glycated haemoglobin, % (s.d.)	8.2 (1.7)	7.9 (1.5)	0.194	8.1 (1.6)	8.1 (1.7)	–0.001
Mean γ-glutamyl transpeptidase, mg/dl (s.d.)	68.1 (68.3)	72.0 (73.1)	–0.055	69.7 (69.8)	69.6 (70.7)	0.001
Proteinuria, *n* (%)			–0.035			0.008
1	10 379 (77)	6066 (76)		10 315 (76)	6119 (77)	
2	1634 (12)	1003 (12)		1637 (12)	964 (12)	
3	1042 (8)	600 (7)		1032 (8)	606 (8)	
4	367 (3)	269 (3)		399 (3)	230 (3)	
5	113 (1)	88 (1)		122 (1)	66 (1)	
Charlson Comorbidity Index, *n* (%)			–0.089			0.006
0	6044 (45)	3231 (40)		5825 (43)	3471 (44)	
1	4078 (30)	2471 (31)		4101 (30)	2409 30)	
2	1788 (13)	1203 (15)		1869 (14)	1109 (14)	
3	742 (5)	559 (7)		802 (6)	467 (6)	
≥4	883 (7)	562 (7)		907 (7)	528 (7)	
Comorbidities, *n* (%)						
Hypertension	6135 (45)	4431 (55)	–0.199	6611 (49)	3902 (49)	0.002
Dyslipidaemia	8046 (59)	5246 (65)	–0.122	8320 (62)	4924 (62)	–0.001
Stroke	287 (2)	182 (2)	–0.01	284 (2)	162 (2)	0.005
Myocardial infarction	83 (1)	153 (2)	–0.116	150 (1)	87 (1)	0.002
Heart failure	594 (4)	710 (9)	–0.18	808 (6)	487 (6)	–0.004
Atrial fibrillation	148 (1)	155 (2)	–0.069	187 (1)	109 (1)	0.002
Venous thromboembolism	65 (0)	56 (1)	–0.028	72 (1)	44 (1)	–0.002
COPD	60 (0)	34 (0)	0.003	57 (0)	32 (0)	0.002
Chronic kidney disease	128 (1)	144 (2)	–0.073	167 (1)	101 (1)	–0.002
Septic arthritis	23 (0)	13 (0)	0.002	17 (0)	11 (0)	–0.003
Pseudogout	22 (0)	23 (0)	–0.026	24 (0)	13 (0)	0.004
RA	366 (3)	201 (3)	0.013	353 (3)	203 (3)	0.005
Trauma	1145 (8)	722 (9)	–0.019	1176 (9)	686 (9)	0.004
Medications, *n* (%)						
Antihyperlipidaemic	5041 (37)	3629 (45)	–0.162	5426 (40)	3218 (40)	–0.002
Insulin	1090 (8)	477 (6)	0.083	976 (7)	583 (7)	–0.003
DPP4 inhibitors	6912 (51)	3810 (47)	0.072	6730 (50)	3971 (50)	0.002
GLP-1 receptor agonists	142 (1)	96 (1)	–0.014	142 (1)	85 (1)	–0.001
Sulfonylureas	1507 (11)	843 (11)	0.02	1472 (11)	862 (11)	0.003
Other hyperglycaemic drugs	1537 (11)	932 (12)	–0.008	1552 (11)	930 (12)	–0.005
Antiplatelet drugs	639 (5)	651 (8)	–0.139	804 (6)	462 (6)	0.007
Anticoagulant drugs	94 (1)	92 (1)	–0.047	106 (1)	63 (1)	0.000
Cardiac glycosides	19 (0)	16 (0)	–0.014	13 (0)	7 (0)	0.003
Antiarrhythmic drugs	46 (0)	56 (1)	–0.05	65 (0)	37 (0)	0.002
Diuretic drugs	436 (3)	427 (5)	–0.104	529 (4)	324 (4)	–0.007
Beta-blockers	647 (5)	731 (9)	–0.171	864 (6)	513 (6)	–0.001
Calcium channel blockers	2771 (20)	1917 (24)	–0.082	2933 (22)	1724 (22)	0.003
ACE inhibitor/ARB	3737 (28)	3008 (37)	–0.212	4215 (31)	2490 (31)	0.001
Endocrine therapy	62 (0)	36 (0)	0.001	59 (0)	35 (0)	0.000
Immunosuppressants	64 (0)	47 (1)	–0.016	66 (0)	40 (1)	–0.002
NSAIDs	2190 (16)	1285 (16)	0.005	2179 (16)	1280 (16)	0.003
Systemic corticosteroids	326 (2)	255 (3)	–0.047	361 (3)	215 (3)	–0.001

ACE inhibitor/ARB: angiotensin-converting enzyme inhibitor/angiotensin receptor blocker; COPD: chronic obstructive pulmonary disease; DPP4: dipeptidyl peptidase-4; GLP-1: glucagon-like peptide-1; HDL-C: serum high-density lipoprotein cholesterol; IPTW: inverse probability treatment weighting; LDL-C: low-density lipoprotein cholesterol; NSAIDs: non-steroidal anti-inflammatory drugs; SMD: standardized mean difference; SGLT-2i: sodium–glucose co-transporter-2 inhibitor.

### Primary outcome

**Figure 2.**
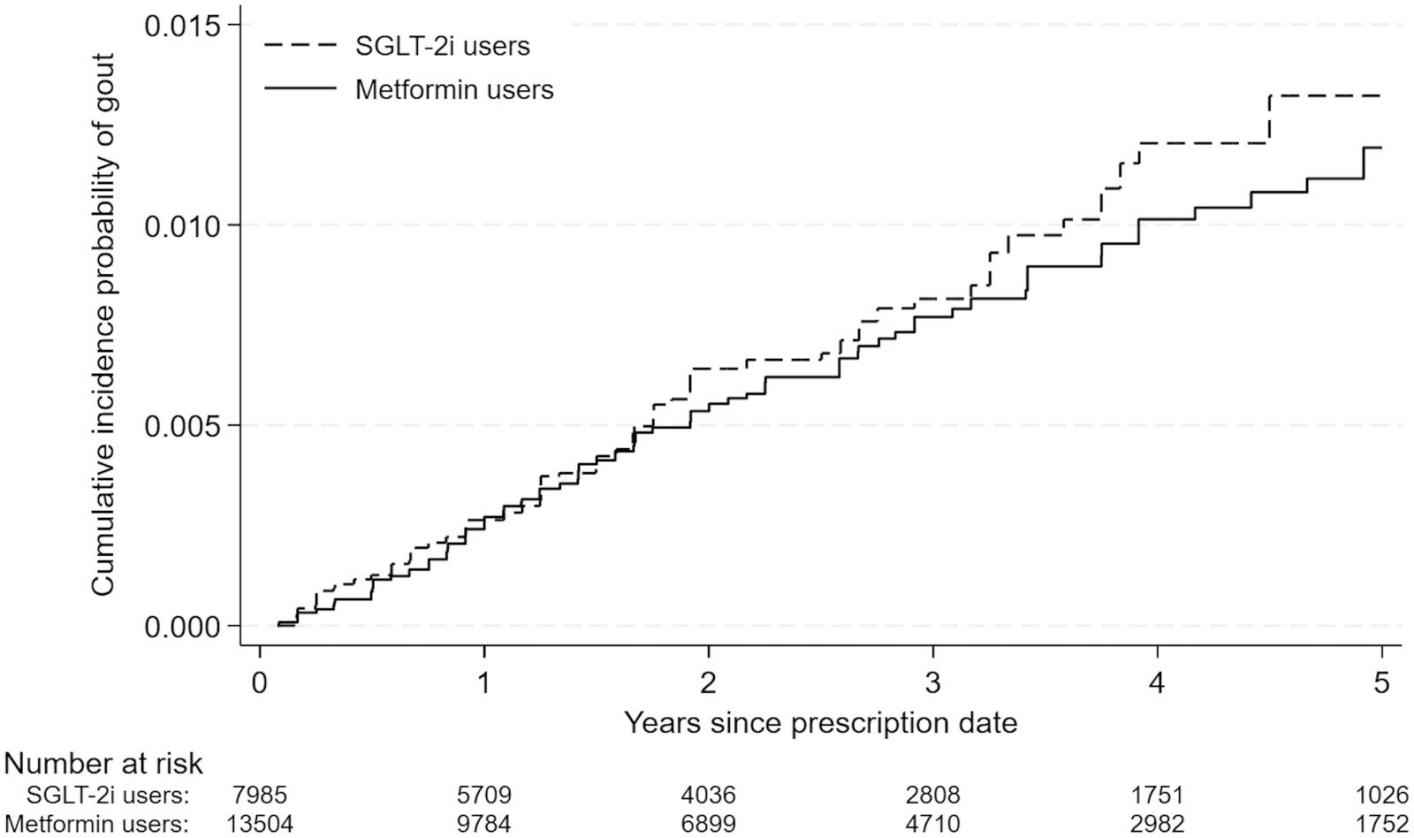
Cumulative incidence probability of gout after inverse probability treatment weighting. SGLT-2i: sodium–glucose co-transporter-2 inhibitor

Table 2 shows the results of primary outcome analyses. Overall, 88 of the 13 535 individuals (incidence rate of 2.40 events per 1000 person-years, 95% CI 1.95–2.96) and 56 of the 8026 individuals (incidence rate of 3.15 events per 1000 person-years, 95% CI 2.42–4.09) who were treated with metformin and SGLT-2is, respectively, had gout (Table 2). After IPTW, the metformin group was not associated with a decreased risk of gout compared with the SGLT-2i group (HR 0.90, 95% CI 0.63–1.28; rate difference –0.28, 95% CI –1.24 to 0.68 events per 1000 person-years) (Table 2). As shown in Fig. 2, fewer differences were observed in the cumulative incidence probability of gout between the metformin and SGLT-2i groups.

**Table 2.** Incidence rates and HRs for incident gout comparing individuals who initiated metformin or SGLT-2is

			Before IPTW		After IPTW		
	Number of individuals	Number of events	IR/1000 person-years (95% CI)	HR (95% CI)	IR/1000 person-years (95% CI)	HR (95% CI)	Rate difference/1000 person-years (95% CI)
SGLT-2i users	8026	56	3.15 (2.42, 4.09)	Reference	2.72 (2.07, 3.65)	Reference	Reference
Metformin users	13 535	88	2.40 (1.95, 2.96)	0.77 (0.55, 1.07)	2.44 (1.98, 3.05)	0.90 (0.63, 1.28)	–0.28 (–1.24, 0.68)

HR: hazard ratio; IPTW: inverse probability treatment weighting; IR: incidence rate; SGLT-2i: sodium–glucose co-transporter-2 inhibitor.

### Secondary outcome

Overall, 9942 (46.1%) non-missing serum uric acid data were paired from the first to the second health check-up (Supplementary Table S8, available at *Rheumatology* online). The mean difference in serum uric acid level change was 0.48 mg/dl (95% CI 0.43–0.52) for metformin compared with SGLT-2is, indicating a significant difference in serum uric acid level change, favouring the SGLT-2i group (Supplementary Table S9, available at *Rheumatology* online).

### Subgroup and sensitivity analysis

**Figure 3.**
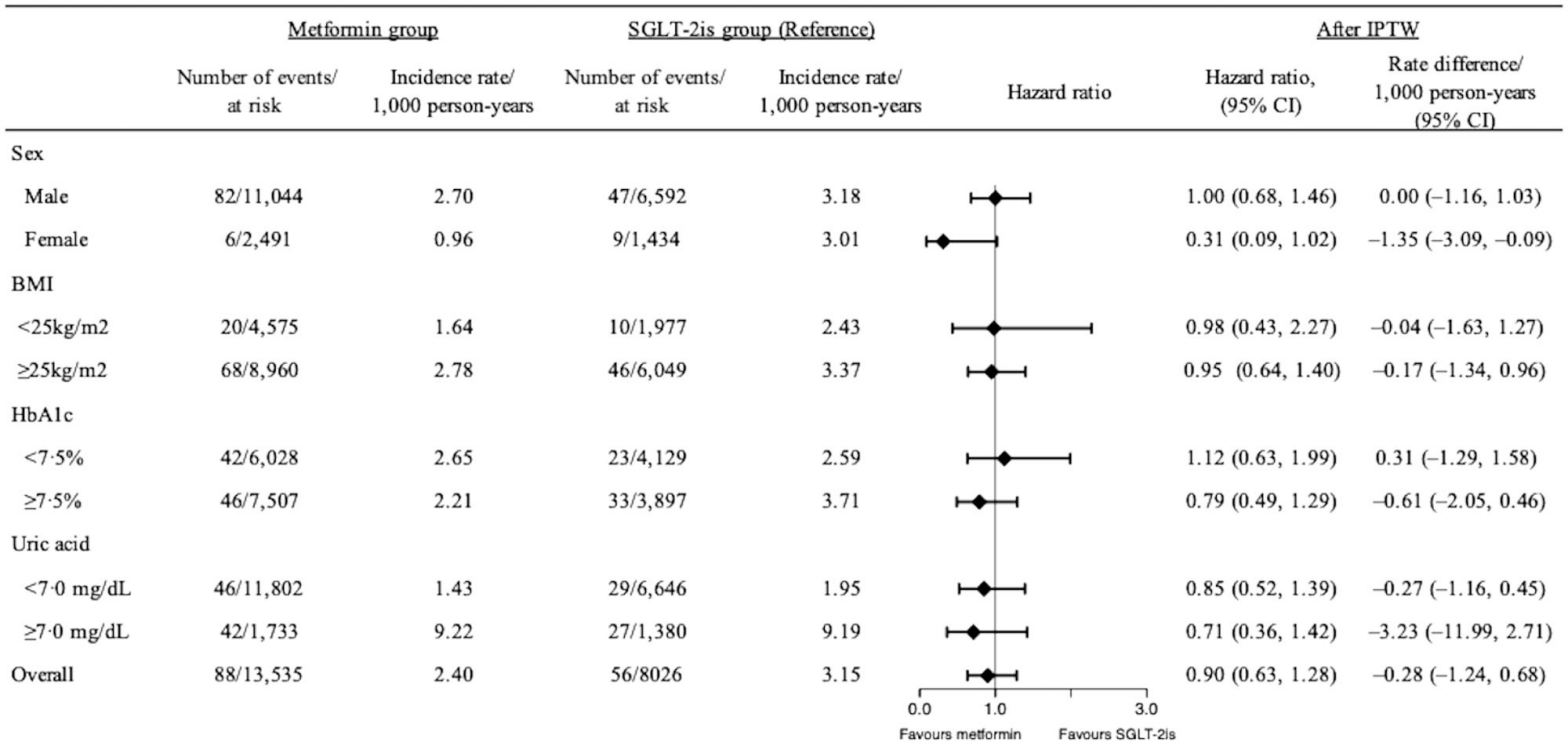
Gout incidence rates and hazard ratios comparing individuals who initiated metformin or SGLT-2is, stratified by sex, BMI, baseline glycated haemoglobin levels and baseline serum uric acid levels. HbA1c: glycated haemoglobin; IPTW: inverse probability treatment weighting; SGLT-2i: sodium–glucose co-transporter-2 inhibitor

In females, the metformin group was associated with a lower risk of incident gout compared with the SGLT-2i group (HR 0.31, 95% CI 0.09–1.02) (*P* = 0.040 for interaction) (Fig. 3). No significant differences were observed in the risk of incident gout between the two groups for males (HR 1.00, 95% CI 0.68–1.46) (Fig. 3). Furthermore, no significant differences were observed in other subgroup outcomes between the two groups (Fig. 3).

Similar trends in the primary outcomes were observed across sensitivity analyses (Supplementary Tables S10–S12, available at *Rheumatology* online).

## Discussion

This new-user comparative effectiveness study that used a nationwide administrative claims database with health check-up data found that metformin and SGLT-2is may be comparable in terms of gout risk among individuals with diabetes. Metformin was less effective than SGLT-2is in lowering serum uric acid levels.

Antidiabetic medications may influence gout risk among individuals with diabetes [8–11, 16, 28]. SGLT-2is have been shown to reduce gout risk in this population [8–11]. However, evidence on the impact of metformin on gout risk is limited. A study showed that metformin use reduced gout risk by 32% in individuals with pre-diabetes (HbA1c level 5.7–6.4%) [16]. Another case-control study found a decreased odds ratio of 0.71 (95% CI 0.65–0.77) for developing gout among individuals with diabetes. However, this study compared metformin with heterogeneous antidiabetic drug use [28]. Our findings provide additional evidence that metformin and SGLT-2is have comparable effectiveness in terms of gout risk in individuals with diabetes with HbA1c levels ≥6.5%.

The serum urate-lowering effects of metformin have shown mixed results [16, 29]. In contrast, SGLT-2is may lower serum uric acid by increasing renal urate elimination, thereby reducing gout risk [7]. Our study’s analysis of serum uric acid level changes was limited due to the incomplete data; however, it suggested that SGLT-2i use might be more effective in lowering serum uric acid levels among individuals with diabetes than metformin use. Compared with SGLT-2is, metformin users did not show an evident decrease in serum uric acid levels from the first to second health check-up (Supplementary Table S9, available at *Rheumatology* online). Although metformin may not reduce serum uric acid levels as effectively as SGLT-2is, metformin and SGLT-2is have comparable effectiveness in terms of gout risk. This suggests that serum uric acid level reduction has less impact on gout risk in this population. Two reasons may explain this observation. First, 38% of the population had poor HbA1c control (≥8%), and these high HbA1c levels may diminish the effectiveness of serum uric acid level reduction [30]. Second, only 14% of this population had asymptomatic hyperuricaemia (≥7.0 mg/dl), and individuals with a history of gout were excluded. Although serum uric acid level control is crucial for gout prevention, its impact may be less pronounced in individuals without baseline hyperuricaemia and a history of gout [30, 31]. Therefore, future studies should investigate whether the reduction of gout risk by these antihyperglycaemic agents is mediated by changes in serum uric acid levels.

This study suggests that metformin and SGLT-2is demonstrated comparable effectiveness in reducing gout risk among individuals with diabetes, with SGLT-2is providing greater reductions in serum uric acid levels. These findings provide valuable guidance for clinicians and patients in addressing glycaemic control and gout prevention. Recent evidence indicates that combining SGLT-2is with metformin can enhance gout prevention compared with metformin alone [32]. This evidence highlights the potential of SGLT-2is as an effective second-line agent, complementing the established role of metformin as a first-line therapy, particularly when gout prevention is a clinical consideration in diabetes management.

This study has several strengths. First, we used an active comparator and new-user design with time-stratified IPTW to mitigate the risk of confounding by indication and unmeasured clinical characteristics. Second, the JMDC database enabled the adjustment of important potential confounders, including medical complications, BMI, blood pressure, prescription history, lifestyle factors and baseline laboratory values, which are typically unavailable in other datasets. Third, we employed robust pharmaco-epidemiological methods. The primary analysis used an observational analogue of initial-treatment approach, providing conservative estimates to define drug use. Additionally, the sensitivity analysis was conducted using an as-treated definition of drug use to evaluate gout risk in individuals, censoring those who discontinued the study drug or received the other study drug during the follow-up. Consistent with the main initial-treatment analysis, the as-treated analysis also revealed a similar risk in individuals who used metformin and those who used SGLT-2is.

Our study had some limitations. First, although the primary outcome was defined using previously validated claims-based algorithms [9, 21], a possibility of outcome misclassification remains. However, such misclassification was unlikely to differ between individuals receiving metformin and those receiving SGLT-2is. Second, many subgroup analyses had a limited number of events, resulting in large CIs. Further accumulation of clinical data on the use of these antihyperglycaemic agents is warranted to enable more robust analyses. Third, the definitions of the outcomes and diseases were not validated. Fourth, in this study, cumulative medication exposure for SGLT-2is and metformin was not incorporated into the analysis due to limitation of data.

In conclusion, this study indicates that the risk of gout associated with metformin is comparable to that of SGLT-2is in individuals with diabetes, with a lesser reduction in serum uric acid levels.

## Supplementary Material

keaf136_Supplementary_Data

## Data Availability

The data for this article were provided by JMDC database, Inc. under licence. Data will be shared by the corresponding author upon request, with permission from JMDC database, Inc.
