# Supplementary material for: Gout incidence in metformin versus sodium–glucose co-transporter-2 inhibitor users: a retrospective cohort study
Source: Rheumatology (Oxford). 2025 Mar 24;64(7):4164–71. doi: 10.1093/rheumatology/keaf136 (PMC12212906; doi:10.1093/rheumatology/keaf136)
Supplement: keaf136_Supplementary_Data [file keaf136_supplementary_data.zip › keaf136_Supplementary_Data/rhe-24-2356-File005.docx]

**Supplemental Material**

**Supplementary Table S1 ICD-10 codes and original Japanese procedure codes for exclusion criteria**

| **Exclusion criteria** | **ICD-10 codes** |
| --- | --- |
| Type 1 diabetes | E10, E100-109 |
| Severe renal impairment | N19, Z49, I120, I131, N185, N186, N250, Z992 |
|  | **Original Japanese procedure codes** |
| Maintenance dialysis | C102, C156, J038 |
| Renal transplantation | K709-05, K780-00, K780-02 |

ICD-10, International Classification of Diseases, 10th Revision.

**Supplementary Table S2 WHO-ATC codes for exclusion criteria**

| **Exclusion criteria** | **WHO-ATC codes** |
| --- | --- |
| Allopurinol | M04AA01 |
| Febuxostat | M04AA03 |
| Probenecid | M04AB01 |
| Benzbromarone | M04AB03 |
| Colchicine | M04AC01 |

WHO-ATC, World Health Organization Anatomical Therapeutic Chemical Classification System.

**Supplementary Table S3 WHO-ATC codes for outcomes**

| **Covariates** | **WHO-ATC codes** |
| --- | --- |
| Nonsteroidal anti-inflammatory drugs | M01A |
| Colchicine | M04AC01 |
| Intra-articular corticosteroid | H02AB01, H02AB02, H02AB04, H02AB08 |
| Oral Corticosteroid | H02AA02, H02AB01, H02AB02, H02AB04, H02AB05, H02AB06, H02AB08, H02AB09, H02AB10, H02BX |

WHO-ATC, World Health Organization Anatomical Therapeutic Chemical Classification System.

**Supplementary Table S4** **ICD-10 codes for covariates**

| **Covariates** | **ICD-10 codes** |
| --- | --- |
| Hypertension | I10, I15 |
| Dyslipidaemia | E78 |
| Rheumatoid arthritis | M05, M06 |
| Heart failure | I50, I099, I110, I130, I132 |
| Atrial fibrillation | I48 |
| Venous thromboembolism | I260, I269, I80, I81, I82 |
| Chronic kidney disease | N18 |
| Septic arthritis | M009 |
| Chronic obstructive pulmonary disease | J44 |
| Pseudogout | M112 |
| Trauma | S4, S5, S6, S7, S8, S9, T1, T10, T11, T12, T13, T14 |

ICD-10, International Classification of Diseases, 10th Revision.

**Supplementary Table S5 WHO-ATC codes for covariates**

| **Covariates** | **WHO-ATC codes** |
| --- | --- |
| Insulin | A10A |
| DPP4 inhibitors | A10BH |
| GLP-1 agonists | A10BJ |
| Sulfonylureas | A10BB |
| Alpha-glucosidase inhibitors fixation | A10BF |
| Thiazolidinediones | A10BG |
| Glinides | A10BX |
| Antiplatelet drugs | B01AC, B01AC04, B01AC05, B01AC06, B01AC19, B01AC22, B01AC23, B01AC30, B01AC56 |
| Anticoagulant drugs | B01AE07, B01AF |
| Cardiac glycosides | C01AA |
| Antiarrhythmic drugs | C01B |
| Betablockers | C07 |
| Calcium channel blockers | C08 |
| ACE-inhibitor/ARB | C09 |
| Antihyperlipidemic drugs | C10 |
| Endocrine therapy | L02 |
| Immunosuppressant drugs | L04 |
| Diuretic drugs | C03 |
| Nonsteroidal anti-inflammatory drugs | M01A |
| Oral corticosteroids | H02AA02, H02AB01, H02AB02, H02AB04, H02AB05, H02AB06, H02AB08, H02AB09, H02AB10, H02BX |

ACE-inhibitor/ARB, angiotensin-converting enzyme inhibitor/angiotensin receptor blockers, DPP4, dipeptidyl peptidase-4; GLP-1, glucagon-like peptide-1.

**Supplementary Table S6 Percentage of missing data for covariates**

| **Covariates** | **Percentage** |
| --- | --- |
| Body mass index | <0.1% |
| Smoking status | 5.5% |
| Alcohol consumption | 7.1% |
| Systolic blood pressure | 0.2% |
| Serum high-density lipoprotein cholesterol | <0.1% |
| Serum triglycerides | 0.2% |
| Serum low-density lipoprotein cholesterol | <0.1% |
| Serum γ-Glutamyl transpeptidase | 0.9% |
| Proteinuria | 1.3% |

**Supplementary Table S7** **Distribution of the censoring events**

|  | **Initial-treatment analysis** | | **As-treated analysis** | |
| --- | --- | --- | --- | --- |
|  | **SGLT-2is** | **Metformin** | **SGLT-2is** | **Metformin** |
| **Censoring reasons (%)** | N = 8,026 | N = 13,535 | N = 8,026 | N = 13,535 |
| End of the study | 6,659 (83%) | 10,687 (79%) | 3,875 (48%) | 4,991 (37%) |
| Administrative censoring | 1,293 (16%) | 2,713 (20%) | 806 (10%) | 1,460 (11%) |
| Treatment discontinuation |  |  | 1,612 (20%) | 3,407 (25%) |
| Add |  |  | 1,558 (19%) | 3,318 (25%) |
| Switch |  |  | 124 (2%) | 266 (2%) |
| Gout outcome | 56 (1%) | 88 (1%) | 33 (0%) | 46 (0%) |
| Death | 18 (0%) | 47 (0%) | 18 (0%) | 47 (0%) |

SGLT-2i, sodium–glucose co-transporter-2 inhibitor

**Supplementary Table S8 Characteristics of individuals who initiated metformin or sodium–glucose co-transporter-2 inhibitors before and after inverse probability treatment weighting, limited to those with serum uric acid levels recorded at the second health check-up**

|  | **Before IPTW** | |  | **After IPTW** | |  |
| --- | --- | --- | --- | --- | --- | --- |
|  | **Metformin group** | **SGLT-2i group** | **SMD** | **Metformin group** | **SGLT-2i group** | **SMD** |
| Total participants, n | n = 6,523 | n = 3,419 |  | n = 6,480 | n = 3,376 |  |
| Mean age, years (SD) | 51.9 (7.9) | 52.0 (8.0) | –0.013 | 51.9 (7.9) | 51.9 (8.0) | –0.002 |
| Male sex, n (%) | 5,480 (84%) | 2,888 (84%) | –0.013 | 5,447 (84%) | 2,836 (84%) | 0.002 |
| Mean BMI, kg/m^2^ (SD) | 27.3 (4.6) | 28.5 (4.8) | –0.252 | 27.7 (4.9) | 27.7 (4.5) | 0.001 |
| Smoking status (current smoker), n (%) | 2,461 (38%) | 1,206 (35%) | –0.051 | 2,389 (37%) | 1,234 (37%) | –0.007 |
| Alcohol consumption (daily), n (%) | 1,384 (21%) | 734 (21%) | 0.006 | 1,374 (21%) | 701 (21%) | –0.010 |
| Mean systolic blood pressure, mmHg (SD) | 131.0 (16.8) | 132.0 (16.6) | –0.059 | 131.4 (16.8) | 131.4 (16.8) | –0.004 |
| Mean uric acid, mg/dL (SD) | 5.6 (1.3) | 5.7 (1.3) | –0.143 | 5.6 (1.3) | 5.6 (1.3) | 0.004 |
| Mean HDL, mg/dL (SD) | 51.3 (13.0) | 51.0 (12.5) | 0.027 | 51.2 (13.0) | 51.3 (12.7) | –0.004 |
| Mean triglycerides, mg/dL (SD) | 183.5 (158.2) | 180.2 (149.1) | 0.021 | 182.2 (153.9) | 181.4 (154.7) | 0.005 |
| Mean LDL, mg/dL (SD) | 133.2 (34.2) | 130.2 (33.1) | 0.089 | 132.1 (34.0) | 132.1 (33.3) | 0.000 |
| Mean hemoglobin A1c, % (SD) | 8.1 (1.6) | 7.9 (1.4) | 0.160 | 8.0 (1.5) | 8.0 (1.5) | 0.014 |
| Mean γ-Glutamyl transpeptidase, mg/dL (SD) | 67.5 (66.6) | 72.0 (75.1) | –0.064 | 69.2 (68.5) | 69.2 (70.8) | 0.000 |
| **Proteinuria, n (%)** |  |  | –0.022 |  |  | 0.013 |
| 1 | 4,946 (76%) | 2,561 (75%) |  | 4,902 (76%) | 2,568 (76%) |  |
| 2 | 836 (13%) | 449 (13%) |  | 834 (13%) | 431 (13%) |  |
| 3 | 496 (8%) | 267 (8%) |  | 507 (8%) | 263 (8%) |  |
| 4 | 182 (3%) | 113 (3%) |  | 186 (3%) | 93 (3%) |  |
| 5 | 63 (1%) | 29 (1%) |  | 50 (1%) | 22 (1%) |  |
| **Charlson Comorbidity Index, n (%)** | |  | –0.075 |  |  | 0.008 |
| 0 | 2,926 (45%) | 1,405 (41%) |  | 2,836 (44%) | 1,491 (44%) |  |
| 1 | 1,961 (30%) | 1,038 (30%) |  | 1,960 (30%) | 1,012 (30%) |  |
| 2 | 876 (13%) | 524 (15%) |  | 908 (14%) | 479 (14%) |  |
| 3 | 374 (6%) | 249 (7%) |  | 399 (6%) | 202 (6%) |  |
| ≥4 | 386 (6%) | 203 (6%) |  | 377 (6%) | 193 (6%) |  |
| **Comorbidities, n (%)** |  |  |  |  |  |  |
| Hypertension | 2,921 (45%) | 1,848 (54%) | –0.186 | 3,096 (48%) | 1,604 (48%) | 0.005 |
| Dyslipidemia | 3,932 (60%) | 2,247 (66%) | –0.113 | 4,020 (62%) | 2,083 (62%) | 0.007 |
| Stroke | 132 (2%) | 62 (2%) | 0.015 | 124 (2%) | 61 (2%) | 0.009 |
| Myocardial infraction | 43 (1%) | 59 (2%) | –0.098 | 67 (1%) | 33 (1%) | 0.007 |
| Heart failure | 284 (4%) | 283 (8%) | –0.162 | 357 (6%) | 180 (5%) | 0.007 |
| Atrial fibrillation | 76 (1%) | 60 (2%) | –0.049 | 79 (1%) | 46 (1%) | –0.013 |
| Venous thromboembolism | 30 (0%) | 22 (1%) | –0.025 | 30 (0%) | 17 (0%) | –0.005 |
| COPD | 25 (0%) | 10 (0%) | 0.016 | 21 (0%) | 10 (0%) | 0.005 |
| Chronic kidney disease | 60 (1%) | 50 (1%) | –0.05 | 69 (1%) | 36 (1%) | –0.001 |
| Septic arthritis | 14 (0%) | 5 (0%) | 0.016 | 6 (0%) | 4 (0%) | –0.007 |
| Pseudogout | 10 (0%) | 8 (0%) | –0.018 | 8 (0%) | 5 (0%) | –0.001 |
| Rheumatoid arthritis | 203 (3%) | 99 (3%) | 0.013 | 193 (3%) | 94 (3%) | 0.011 |
| Trauma | 558 (9%) | 320 (9%) | –0.028 | 570 (9%) | 298 (9%) | –0.001 |
| **Medications, n (%)** |  |  |  |  |  |  |
| Antihyperlipidemic | 2,453 (38%) | 1,521 (44%) | –0.140 | 2,578 (40%) | 1,347 (40%) | –0.002 |
| Insulin | 477 (7%) | 186 (5%) | 0.077 | 422 (7%) | 211 (6%) | 0.010 |
| DPP4 inhibitors | 3,551 (54%) | 1,777 (52%) | 0.049 | 3,486 (54%) | 1,814 (54%) | 0.001 |
| GLP-1 receptor agonists | 66 (1%) | 41 (1%) | –0.018 | 64 (1%) | 33 (1%) | 0.000 |
| Sulfonylureas | 822 (13%) | 459 (13%) | –0.024 | 835 (13%) | 431 (13%) | 0.003 |
| Other hyperglycemic drugs | 868 (13%) | 499 (15%) | –0.037 | 890 (14%) | 465 (14%) | –0.001 |
| Antiplatelet drugs | 309 (5%) | 252 (7%) | –0.111 | 359 (6%) | 179 (5%) | 0.01 |
| Anticoagulant drugs | 47 (1%) | 35 (1%) | –0.033 | 46 (1%) | 25 (1%) | –0.002 |
| Cardiac glycosides | 12 (0%) | 9 (0%) | –0.017 | 5 (0%) | 2 (0%) | 0.004 |
| Antiarrhythmic drugs | 26 (0%) | 16 (0%) | –0.011 | 22 (0%) | 10 (0%) | 0.006 |
| Diuretic drugs | 208 (3%) | 149 (4%) | –0.061 | 221 (3%) | 115 (3%) | 0.000 |
| Betablockers | 325 (5%) | 295 (9%) | –0.145 | 398 (6%) | 196 (6%) | 0.013 |
| Calcium channel blockers | 1,255 (19%) | 759 (22%) | –0.073 | 1,301 (20%) | 668 (20%) | 0.007 |
| ACE inhibitor/ARB | 1,827 (28%) | 1,297 (38%) | –0.212 | 2,027 (31%) | 1,052 (31%) | 0.002 |
| Endocrine therapy | 24 (0%) | 12 (0%) | 0.003 | 23 (0%) | 12 (0%) | 0.001 |
| Immunosuppressants | 22 (0%) | 10 (0%) | 0.008 | 18 (0%) | 10 (0%) | –0.004 |
| NSAIDs | 1,099 (17%) | 585 (17%) | –0.007 | 1,093 (17%) | 564 (17%) | 0.004 |
| Systemic corticosteroids | 151 (2%) | 101 (3%) | –0.040 | 161 (2%) | 84 (2%) | 0.000 |

ACE inhibitor/ARB, angiotensin-converting enzyme inhibitor/angiotensin receptor blocker; BMI, body mass index; COPD, chronic obstructive pulmonary disease; DPP4, dipeptidyl peptidase-4; GLP-1, glucagon-like peptide-1; HDL-C, serum high-density lipoprotein cholesterol; IPTW, inverse probability treatment weighting; LDL-C, low-density lipoprotein cholesterol; NSAIDs, Non-Steroidal Anti-Inflammatory Drugs; SD, standard deviation; SMD, standardised mean difference; SGLT-2i, sodium–glucose co-transporter-2 inhibitor.

**Supplementary Table S9 Marginal predicted serum uric acid levels at the first and second health check-ups, and difference in uric acid change, comparing individuals who initiated metformin vs those who initiated sodium–glucose co-transporter-2 inhibitors**

|  | **Marginal predicted serum uric acid level, mg/dL (SD)** | | **Difference in uric acid change,**  **mg/dL (95%CI)** |
| --- | --- | --- | --- |
|  | **First check-up** | **Second check-up** |  |
| SGLT-2i users | 5.73 (0.02) | 5.28 (0.02) | Reference |
| Metformin users | 5.56 (0.02) | 5.58 (0.02) | 0.48 (0.43, 0.52) |

CI, confidence interval; SD, standard deviation; SGLT-2i, sodium-glucose co-transporter-2 inhibitors.

**Supplementary Table S10 Gout incidence rates and hazard ratios limited to the comparison of individuals who initiated metformin with those who initiated sodium–glucose co-transporter-2 inhibitors, censoring those who discontinued the study drug or received the other study drug during follow-up (as-treated approach)**

|  |  |  | **Before IPTW** | **After IPTW** | |  |
| --- | --- | --- | --- | --- | --- | --- |
|  | **No. of events** | **Incidence rate/1,000 person-years**  **(95% CI)** | **Hazard ratio,**  **(95% CI)** | **Hazard ratio,**  **(95% CI)** | **Rate difference/1,000 person-years (95% CI)** | |
| **SGLT-2i users** | 33 | 2.95 (2.10, 4.15) | Reference | Reference | Reference | |
| **Metformin users** | 46 | 2.28 (1.71, 3.04) | 0.78 (0.50, 1.22) | 0.90 (0.56, 1.44) | –0.28 (–1.61, 0.91) | |

CI, confidence interval; IPTW, inverse probability treatment weighting; SGLT-2i, sodium–glucose co-transporter-2 inhibitor.

**Supplementary Table S11 Gout incidence rates and hazard ratios comparing individuals who initiated metformin with those who initiated sodium–glucose co-transporter-2 inhibitors, with multiple imputation for missing data**

|  |  |  |  | **Before IPTW** | **After IPTW** | |  |
| --- | --- | --- | --- | --- | --- | --- | --- |
|  | **No. of individuals** | **No. of events** | **Incidence rate/1,000 person-years**  **(95% CI)** | **Hazard ratio,**  **(95% CI)** | **Hazard ratio,**  **(95% CI)** | **Rate difference/1,000 person-years (95% CI)** | |
| **SGLT-2i users** | 8,808 | 63 | 3.17 (2.47, 4.05) | Reference | Reference | Reference | |
| **Metformin users** | 15,030 | 107 | 2.58 (2.13, 3.11) | 0.82 (0.60, 1.11) | 0.94 (0.68, 1.32) | –0.16 (–1.12, 0.73) | |

CI, confidence interval; IPTW, inverse probability treatment weighting; SGLT-2i, sodium–glucose co-transporter-2 inhibitor.

**Supplementary Table S12 Hazard ratios for the primary outcome comparing individuals who initiated metformin with those who initiated sodium–glucose co-transporter-2 inhibitors after propensity-score overlap weighting**

|  | **Hazard ratio, (95% CI)** | **Rate difference/1,000 person-years (95% CI)** |
| --- | --- | --- |
| **Gout** |  |  |
| SGLT-2i group | Reference | Reference |
| Metformin group | 0.87 (0.61, 1.24) | –0.38 (-1.40, 0.57) |

CI, confidence interval; SGLT-2i, sodium-glucose co-transporter-2 inhibitor.
