# Supplementary figures and images for: Gout incidence in metformin versus sodium–glucose co-transporter-2 inhibitor users: a retrospective cohort study
Source: Rheumatology (Oxford). 2025 Mar 24;64(7):4164–71. doi: 10.1093/rheumatology/keaf136 (PMC12212906; doi:10.1093/rheumatology/keaf136)

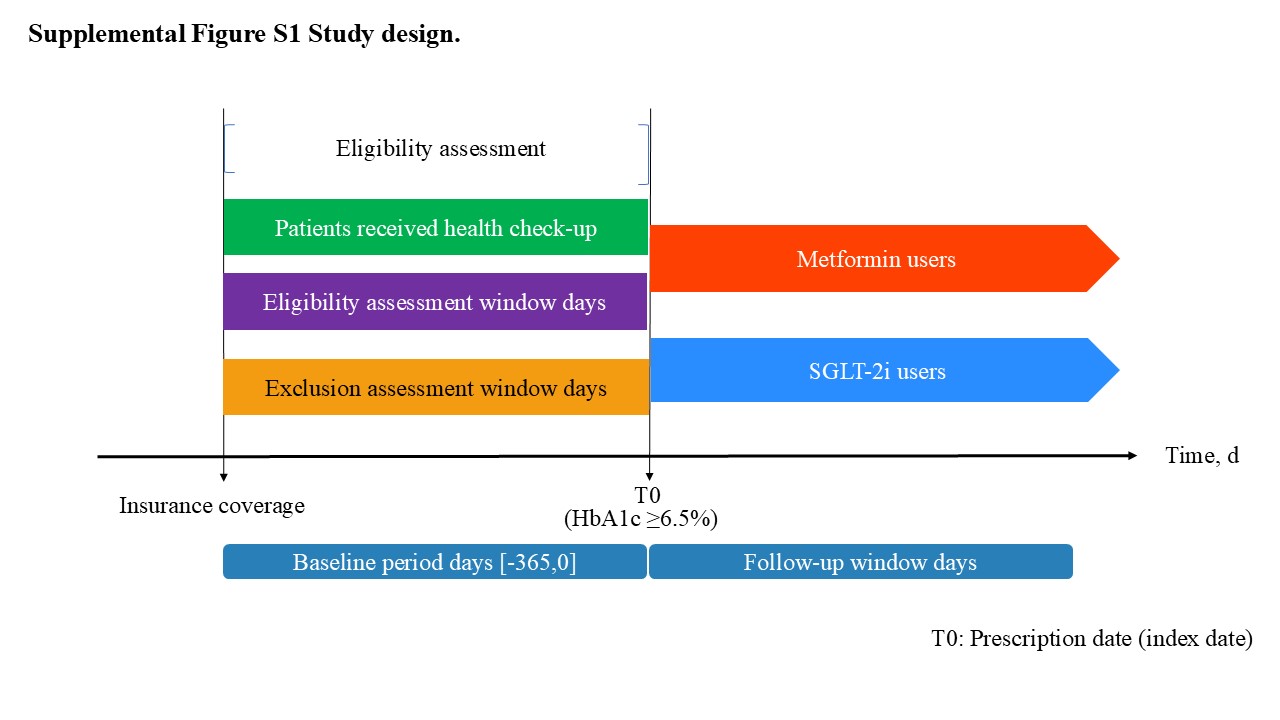

Supplement: keaf136_Supplementary_Data [file keaf136_supplementary_data.zip › keaf136_Supplementary_Data/rhe-24-2356-File006.jpg]
